# Beyond Membranes: Recent Advances in Membrane‐Free Fuel Cells

**DOI:** 10.1002/tcr.202500019

**Published:** 2025-09-04

**Authors:** Meilyn Sanabria León, Maximina Luis‐Sunga, Nicolás Alejandro Sacco, Ilaria Gamba, Gonzalo García

**Affiliations:** ^1^ Instituto Universitario de Materiales y Nanotecnología Departamento de Química Universidad de La Laguna (ULL) PO Box 456 La Laguna 38200 Santa Cruz de Tenerife España; ^2^ Instituto de Investigaciones en Catálisis y Petroquímica INCAPE (UNL‐CONICET) Facultad de Ingeniería Química Santiago del Estero 2829 Santa Fe 3000 Argentina

**Keywords:** fuel cells, membraneless laminar flow fuel cells, mixed‐reactant membraneless fuel cells, polymeric membranes, proton exchange membrane fuel cells

## Abstract

As energy demand increases and the need for sustainable solutions grows, fuel cells have emerged as a promising solution, capable of converting chemical energy into electricity in a clean and combustion‐free process. This technology not only improves energy efficiency but also leads to significant emission reductions, paving the way for a cleaner future. Among the various fuel cell technologies, proton‐exchange membrane fuel cells (PEMFCs) have been at the forefront (Abdelkareem et al., *Sci. Total Environ.* **2021**, *752*, 141803). However, their broader adoption is hindered by key challenges, including high costs, slow reaction kinetics, and internal resistance, which limit their scalability. In response to these challenges, membraneless fuel cells (MFCs) have emerged as an exciting alternative to polymeric membrane systems. By removing the membrane, the system becomes simpler, improving efficiency and robustness, while offering advantages like lower production and maintenance costs, increased durability, and better resistance to chemical degradation. This review focuses on recent advances in the design and catalysis optimization of MFCs. These advancements offer promising avenues for the widespread adoption of MFCs in sustainable energy applications.

## Graphical abstract

Membraneless fuel cells (MFCs) are a promising alternative to conventional membrane‐based systems, offering simplified architectures, reduced costs, and enhanced chemical robustness. By eliminating the polymer membrane, MFCs avoid internal resistance and degradation. This review highlights advances in MFC design and electrocatalysis, emphasizing how they contribute to improved performance, greater durability, and broader applicability in sustainable energy technologies.

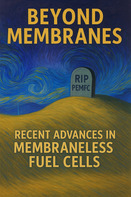

## 1 Introduction

**Figure 1:**
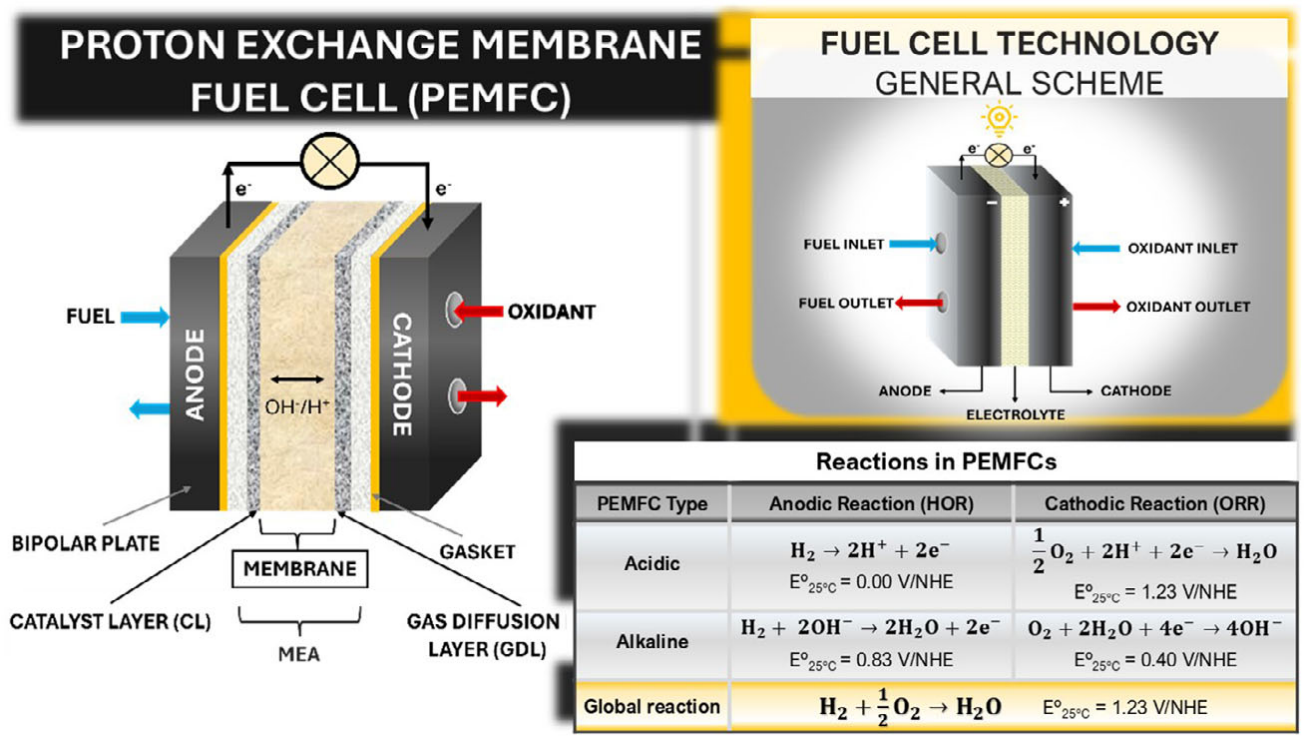
Diagram of general fuel cell technology, PEMFC system, and reactions in acidic and alkaline media.

Fuel cells (FCs) are devices that convert chemical energy directly into electrical energy through electrochemical reactions.^[^ ^1^ ^]^ Unlike traditional combustion systems that rely on fuel combustion and heat release, FCs generate electricity more efficiently, with reduced waste and significantly lower emissions, making them a key technology for mitigating environmental impact.^[^ 2, 3 ^]^ A typical FC system consists of three main components: the anode, cathode, and electrolyte, as illustrated in **Figure** **1**. In proton‐exchange membrane FCs (PEMFCs), these components are part of the membrane‐electrode assembly (MEA), which includes the anode, cathode, and the proton exchange membrane (PEM). The PEM selectively allows protons (H^+^) to migrate from the anode to the cathode while blocking electrons, which are instead directed through an external circuit to generate electric current.^[^ 4, 5 ^]^

At the anode, the hydrogen oxidation reaction (HOR) takes place: hydrogen molecules (H_2_) split into protons and electrons. The protons travel through the PEM to the cathode, where they recombine with electrons and oxygen (O_2_) in the oxygen reduction reaction (ORR), producing water. In contrast, alkaline FCs use a hydroxide‐conducting membrane. In these systems, hydrogen reacts with hydroxide ions at the anode to form water and release electrons, while at the cathode, oxygen reacts with water and electrons to regenerate hydroxide ions. Despite differences in electrolyte type and ionic species, the underlying electrochemical processes are fundamentally similar across both systems.^[^ 6, 7 ^]^

The efficient operation of FCs relies heavily on the use of catalysts to accelerate these reactions at practical rates. In PEMFCs, platinum‐based catalysts remain the standard due to their high activity; however, their cost significantly increases the overall system price.^[^ ^5^ ^]^ Nafion, the most widely used PEM material, also contributes substantially to cost, with prices reaching approximately $2000 per square meter depending on specifications.^[^ ^8^ ^]^ This membrane is complex to manufacture and highly sensitive to hydration levels, factors that limit its practical scalability. In fact, membrane fabrication alone accounts for over 50% of the total cell cost.^[^ ^9^ ^]^ Beyond price, the polymeric nature of PEMs introduces significant internal resistance, particularly at intermediate current densities, which leads to efficiency losses and complicates system optimization. Additional challenges such as fuel crossover, membrane drying, and cathode flooding further hinder the performance and reliability of PEMFCs.^[^ ^10^ ^]^ Overcoming these limitations is essential to reduce costs and enable the large‐scale adoption of FC technology.

Recent research highlights the potential of membraneless FCs (MFCs) as an alternative platform with the capability to address several of these limitations.^[^ ^11^ ^]^ By eliminating the membrane, MFCs reduce production costs, simplify system design, and improve efficiency, making them an attractive option for clean energy production and other applications. Furthermore, MFCs offer increased operational flexibility, which is essential for meeting global energy demands. They overcome many of the limitations associated with PEMFCs, particularly issues related to membrane dehydration, high costs, and restricted temperature ranges.^[^ ^12^ ^]^

**Figure 2:**
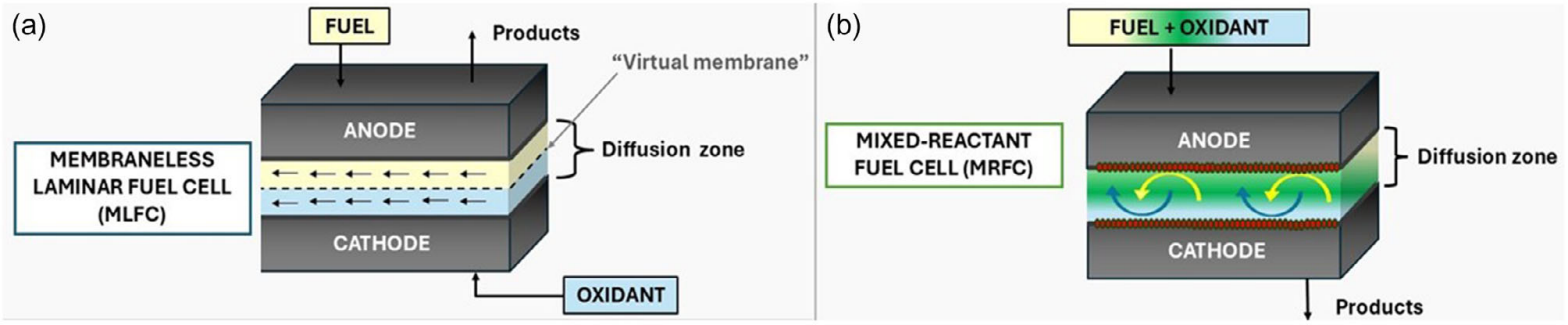
Schematic diagram of the operating principle of a) MLFC and b) MRFC.

MFCs are generally categorized into two types: membraneless laminar flow cells (MLFCs) and mixed reactant FCs (MRFCs), each offering distinct benefits. MLFCs (**Figure** **2**a) utilize microfluidic channels that create parallel flows of fuel and oxidant, relying on laminar flow properties to maintain separation without physical barriers. MRFCs (Figure 2b), by contrast, use a mixed‐reactant stream and achieve selective reaction control through the use of specialized catalysts and porous electrodes that facilitate reactant diffusion and electrochemical selectivity.^[^ ^13^ ^]^ MRFCs rely on catalyst selectivity and electrode architecture to direct the reactions. Both approaches aim to eliminate the cost, complexity, and limitations of polymeric membranes, making MFCs attractive alternatives for compact, efficient, and low‐cost power generation.

**Figure 3:**
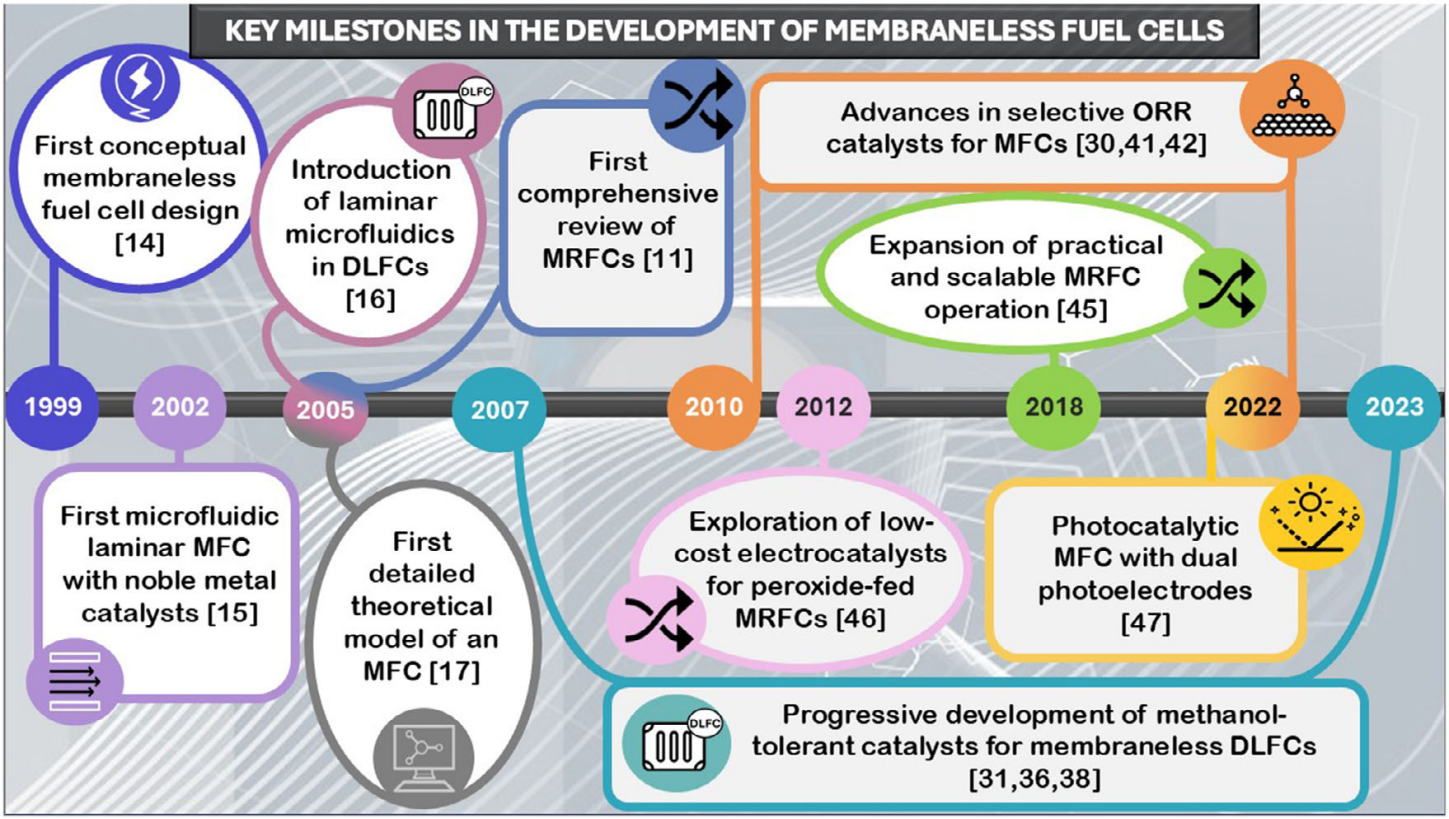
A visual timeline reflecting key scientific progress in the field of MFCs.

The development of MFCs has been shaped by multiple foundational advances, as illustrated in **Figure** **3**. These include the first membraneless configuration proposed by Zhu et al.,^[^ ^14^ ^]^ the first application of laminar microfluidics for reactant separation by Ferrigno et al.,^[^ ^15^ ^]^ the first dual‐electrolyte laminar FC demonstrated by Choban et al.,^[^ ^16^ ^]^ and the first full theoretical model introduced by Ramos,^[^ ^17^ ^]^ among others. These early breakthroughs laid critical groundwork for subsequent innovations in both MLFC and MRFC technologies.

## 2 MLFCs

MLFCs represent a novel approach to traditional FC designs by removing the need for polymer membranes. This simplification reduces manufacturing costs while overcoming the inherent physical limitations of PEMFCs. By controlling the flow of reactants and minimizing convective mixing, MLFCs efficiently separate the fuel and oxidant through diffusion at the interface, effectively creating a “virtual membrane” that allows the electrochemical reactions to occur without a solid polymer membrane. Optimizing MLFC performance relies heavily on careful design. Factors such as strategic electrode placement, the management of diffusive mixing zones, and the selection of flow electrodes are crucial for minimizing issues like reagent crossover and ohmic losses, all of which directly impact efficiency.^[^ ^12^ ^]^ A variety of flow configurations have been explored to enhance reactant separation and improve overall system performance. For instance, Hanapi^[^ ^18^ ^]^ provided a comprehensive review of multiple flow configurations—Y, T, F, H, L, and bridge‐shaped designs—each with distinct advantages.

**Figure 4:**
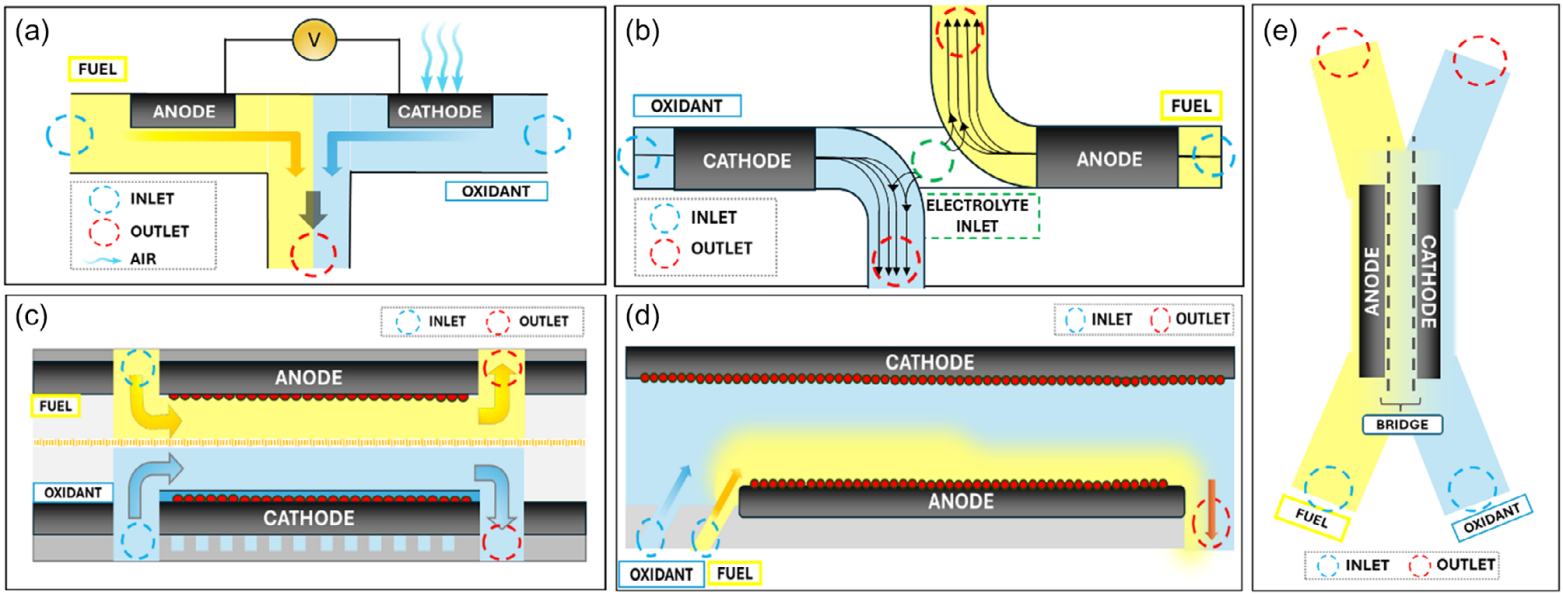
a) Horizontal configuration of T‐shaped MFC, b) L‐shaped membrane less FC, c) H‐shaped membrane less fuel, d) F‐shaped membrane less FC, and e) bridge‐shaped membrane less FC. Adapted from ref. [18], published under a Creative Commons Attribution License (CC BY).

**Figure 5:**
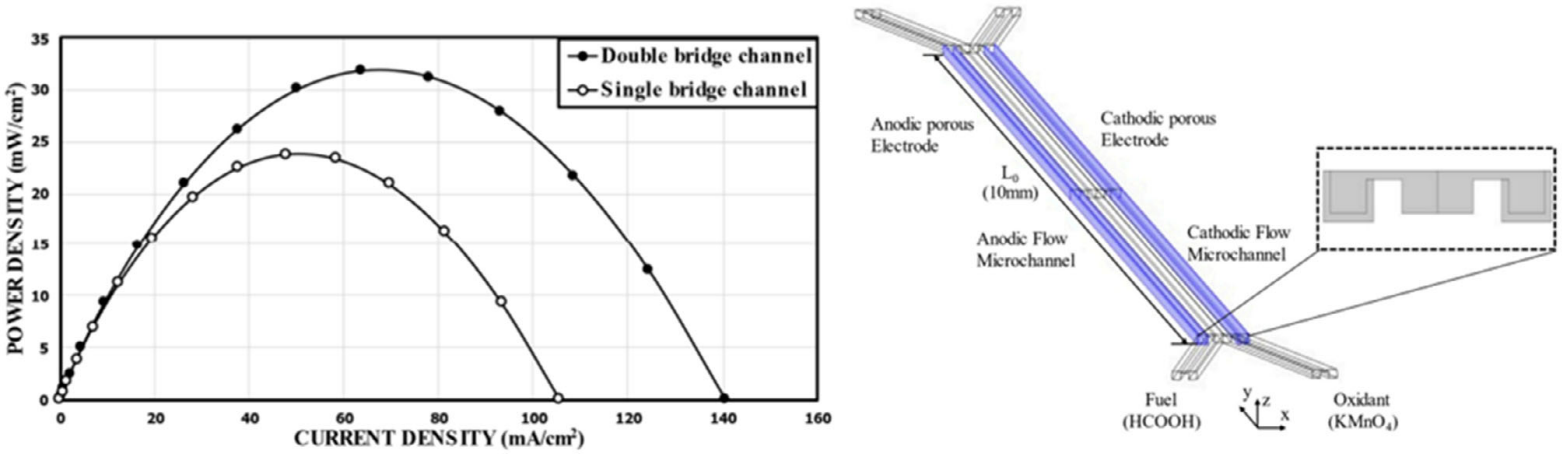
Comparison of predicted performance curves between a single‐bridge channel and the double‐bridge channel. A schematic of the double‐bridge channel is also shown. Adapted from ref. [19], published under a Creative Commons Attribution License (CC BY).

The T‐shape enhances efficiency (**Figure** **4**a), the L‐shape improves fuel utilization (Figure 4b), the H‐shape reduces reactant mixing (Figure 4c), the F‐shape maximizes fuel utilization (Figure 4d), and the bridge‐shape optimizes reactant distribution (Figure 4e). A notable improvement came from Oh et al.,^[^ ^19^ ^]^ who introduced a double‐bridge channel configuration, which achieved a peak power density of 1.65 compared to 1.23 mW cm^−2^ for the traditional single‐bridge design, resulting in a 34% increase in performance (**Figure** **5**). This enhancement was attributed to better control over mixing layers and more efficient transport within the channels. The study also emphasized that optimizing bridge height could further improve performance as setting the bridge height to 90 percent of the channel height raised the power density to 3.33 mW cm^−2^ which represents a 171% increase with respect to the same single‐bridge baseline.

Beyond geometrical configurations, novel designs are emerging to further optimize FC performance. For example, a dual‐electrolyte aluminum/air microfluidic MLFC was developed with a T‐design structure.^[^ ^20^ ^]^ This system features separate alkaline anolyte and acidic catholyte electrolytes, preventing neutralization reactions and boosting electrochemical performance. A carbon paper separator between the two electrolytes minimizes mixing while enabling efficient electrolyte recirculation, making this design particularly promising for portable and cost‐effective power sources.

**Figure 6:**
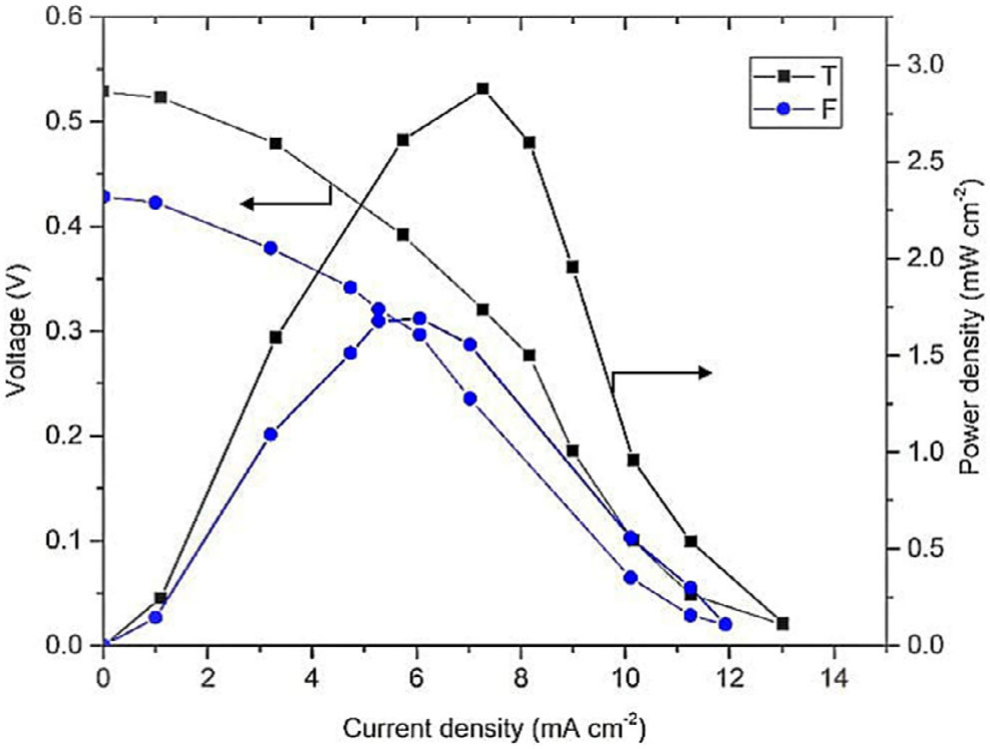
Polarization curve of F and vertically T‐shaped design of membrane‐less DMFC operated at 1 M methanol and 0.5 M of sulfuric acid in open‐air condition. Reproduced from ref. [19], published under a Creative Commons Attribution License (CC BY).

Research into various flow configurations, including F‐shaped and T‐shaped designs,^[^ ^21^ ^]^ has shown significant improvements in MLFCs. The T‐shape design, by reducing the distance between electrodes, helps to decrease ohmic resistance, thereby enhancing the system's efficiency (**Figure** **6**). Moreover, this study demonstrated that the electrocatalysis process plays a critical role in optimizing the ORR. The researchers found that the addition of H_2_O_2_ at the cathode significantly improved the ORR, increasing electrochemical efficiency. Statistical methods such as central composite design (CCD) and response surface methodology (RSM) were used to determine optimal concentrations of reactants, including H_2_O_2_, H_2_SO_4_, and KOH, which resulted in a significant increase in power density. This approach minimizes the risk of over‐saturating the active sites of the electrodes, which could negatively affect performance. By balancing the concentrations of these reactants, the researchers achieved a significant increase in the power density of the cell, demonstrating how the combination of advanced modelling techniques and careful reactant selection can substantially improve the performance of membrane‐less direct methanol FCs (DMFCs).

**Figure 7:**
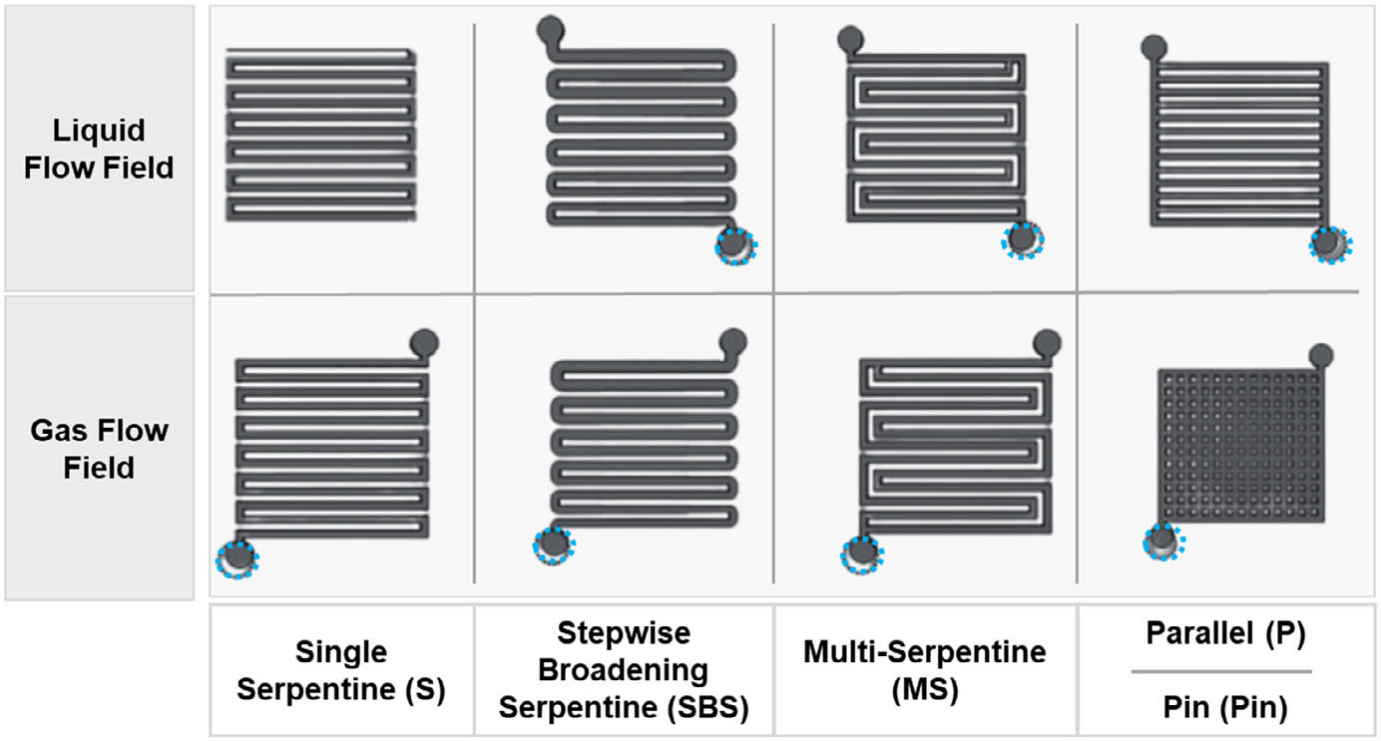
Flow configurations for formic acid MFCs, including single serpentine (S), gradually expanded serpentine (SBS), multiple serpentine (MS), and parallel channels (PIN). The inlet is indicated by a blue circle. Adapted from ref. [19], published under a Creative Commons Attribution License (CC BY).

In another study, Shyu^[^ ^22^ ^]^ examined the impact of flow field designs (see **Figure** **7**) in formic acid MLFCs, emphasizing that air‐breathing MLFCs achieve optimal efficiency when the fuel and electrolyte flow fields are identical. For air‐fed MLFCs, the combination of stepwise broadening serpentine (SBS) liquid flow and multiserpentine (MS) air flow enhances efficiency. A noteworthy result from this study was the “Pin” air flow configuration, where the channel surrounds small holes instead of following a serpentine path. This design improved air distribution and balanced power generation with the energy consumed by the air pump, leading to better overall performance. This analysis highlights how flow field designs can improve the efficiency of MLFCs, which could be crucial for their application in portable energy devices. This study reinforces that, in MLFCs, flow field configuration plays a critical role by promoting uniform fuel distribution, improving CO_2_ removal, and reducing reactant crossover due to the parallel laminar streams. Designs such as SBS and single serpentine are particularly effective in optimizing the electrochemical interface and maintaining efficient operation.

However, in other MFC configurations such as mixed‐reactant FCs (MRFCs), where fuel and oxidant are premixed and enter a shared reaction zone, the influence of flow field geometry on performance is considerably less significant. Since there is no spatial separation between streams and the reactions occur based on selective electrocatalysis, channel configuration mainly affects hydrodynamic resistance rather than reactant management or interface definition.

The analysis of structural design is fundamental for the advancement of membrane‐free systems. However, the development of electrocatalysts plays a crucial role, as their efficiency directly impacts the electrochemical reactions within the system. By improving electrocatalysts, the reaction rate can be optimized, and the activation energy of key processes, such as fuel oxidation and oxygen reduction, can be reduced. This improves overall efficiency, reduces energy losses, and lowers production costs. These advances would not only make MFC systems more effective but also facilitate their adoption in broader commercial applications.

### 2.1 Recent Advances in Electrocatalysis for MLFCs

Electrocatalysis is central to the performance of MLFCs, as it governs the electrochemical reactions that convert chemical energy into electricity. Without a polymer membrane to separate the fuel and oxidant streams, the role of electrocatalysts becomes even more critical. The efficiency and stability of the system depend not only on the catalyst material but also on the electrode configuration, which together influence power output and reaction kinetics.

The nature of the fuel used further affects catalyst behavior, since different fuels present distinct challenges such as poisoning, incomplete oxidation, or gradual performance loss. Addressing these issues requires selecting materials that are chemically compatible with the fuel and capable of sustaining high activity over time. Recent efforts have therefore focused on developing catalysts that combine durability, selectivity, and cost‐effectiveness under realistic operating conditions. These advancements have led to the emergence of three main categories of electrocatalysts for MLFCs: noble metal‐based, transition metal‐based, and hybrid catalysts.

#### 2.1.1 Noble Metal‐Based Catalysts for MLFCs

Noble metals, particularly platinum (Pt), palladium (Pd), and gold (Au), are widely used in MLFCs due to their high catalytic activity, stability, and compatibility with a broad range of electrochemical reactions, including fuel oxidation and oxygen reduction. Their performance under the low‐temperature, colaminar conditions typical of MLFCs makes them key materials in the development of efficient, compact, and stable systems. Recent studies have highlighted how the integration of these catalysts into optimized electrode structures can significantly enhance overall cell performance.

**Figure 8:**
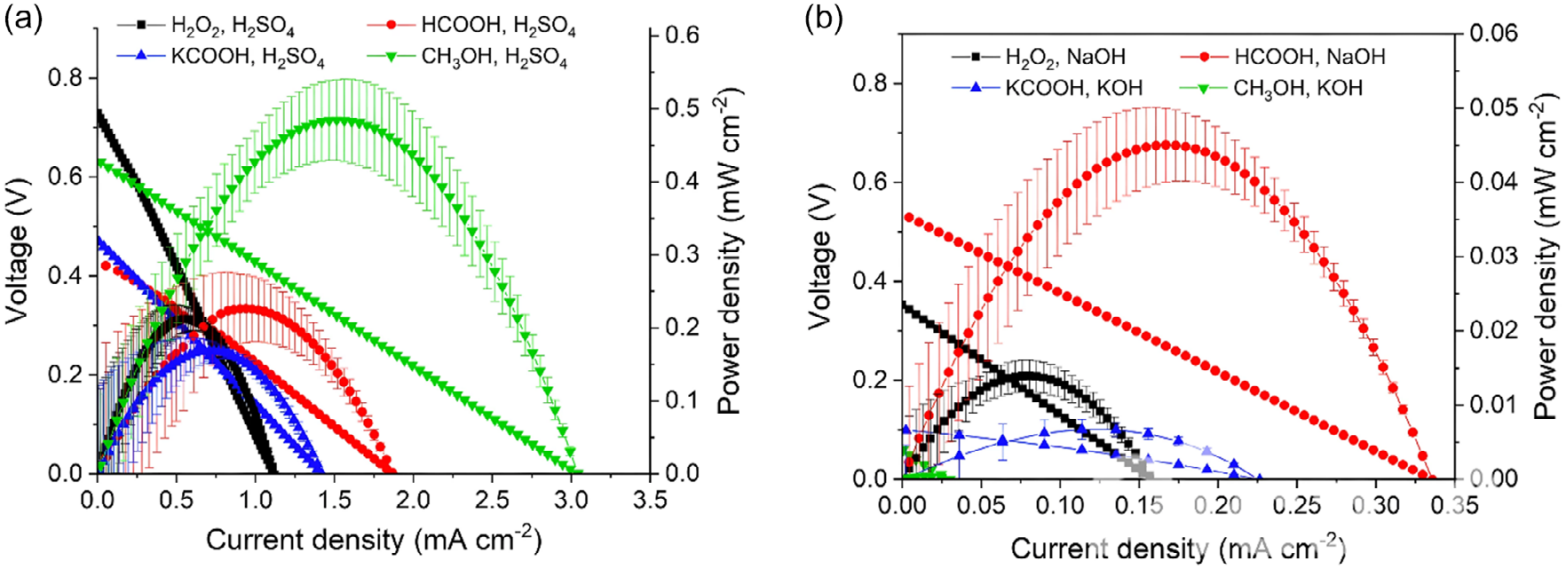
Polarization and power density curves obtained for different 1.0 M fuels using a) acidic and b) alkaline 0.5 M electrolytes. Reproduced with permission.^[^ ^23^ ^]^ Copyright 2023, Elsevier.

One such example is the use of Pt/C and Pd/C supported on a flexible 100% cotton flannel substrate in a microfluidic MLFC design.^[^ ^23^ ^]^ This porous, absorbent material enables passive fuel transport via capillary action, eliminating the need for external pumps and allowing a more uniform and continuous delivery of reactants to the catalytic sites. As a result, the system achieved enhanced mass transport, improved fuel utilization, and increased overall efficiency. This study illustrates the importance of substrate selection and electrode design in maximizing the performance of noble metal catalysts, especially in compact or portable applications. As shown in **Figure** **8**, among the fuel–electrolyte combinations tested in the micro‐MFC, the methanol–sulfuric acid pair exhibited the best performance, reaching 0.48 mW cm^−2^ at 3.05 mA cm^−2^. This outcome is consistent with the known electrocatalytic efficiency of Pt/Ru for methanol oxidation. Notably, acidic electrolytes consistently outperformed alkaline ones, despite the general advantage of ORR kinetics in basic media. This suggests that oxygen solubility, which is higher in acidic solutions, plays a decisive role in microscale membraneless systems where the cathodic oxygen may be supplied by both the atmosphere and the liquid phase. Such results emphasize the critical interplay between fuel type, catalyst composition, and electrolyte environment in optimizing MFC performance.

Building on the established activity of Pt and Pd individually, bimetallic systems have been investigated to exploit synergistic effects that can further improve electrooxidation efficiency. Panjiara and Pramanik^[^ ^24^ ^]^ tested Pd–Pt/C catalysts for glycerol oxidation. Although their approach followed a conventional microfluidic configuration, the results provided valuable validation of the enhanced CO tolerance and catalytic activity afforded by the Pd–Pt combination. While the work did not introduce novel mechanisms or architectures, it reinforced the relevance of Pd–Pt systems in alcohol‐based MLFCs and contributed to consolidating existing trends in noble metal catalysis under realistic operating conditions.

In parallel, alternative noble metals like gold have been studied primarily for their structural and conductive properties rather than their catalytic activity. A noteworthy example is the use of gold‐electroplated carbon fiber cloth electrodes in MLFCs operating with hydrogen peroxide as the oxidant.^[^ ^25^ ^]^ Here, the 3D porous structure of the electrode significantly increased the active surface area and facilitated improved reactant diffusion, resulting in enhanced electrochemical reaction rates and higher power output. Nevertheless, the study also highlighted critical fabrication constraints, noting that excessive electroplating reduced porosity and hindered overall performance. Furthermore, it demonstrated that operational parameters—such as temperature, H_2_O_2_ concentration, flow rate, and electrolyte type—must be carefully tuned to maintain optimal conditions for catalyst activity and system stability.

Taken together, these studies illustrate that the success of noble metal catalysts in MLFCs is not determined solely by their chemical composition but rather by the interplay between material properties, electrode design, and system‐level variables. From flexible substrates to bimetallic and structured electrode designs, each innovation contributes to overcoming limitations in mass transport, durability, or fuel utilization. Although the high cost of noble metals remains a challenge, their superior catalytic performance and long‐term stability continue to make them indispensable in MLFC applications where efficiency and reliability are priorities.

#### 2.1.2 Transition Metal‐Based Catalysts for MLFCs

**Figure 9:**
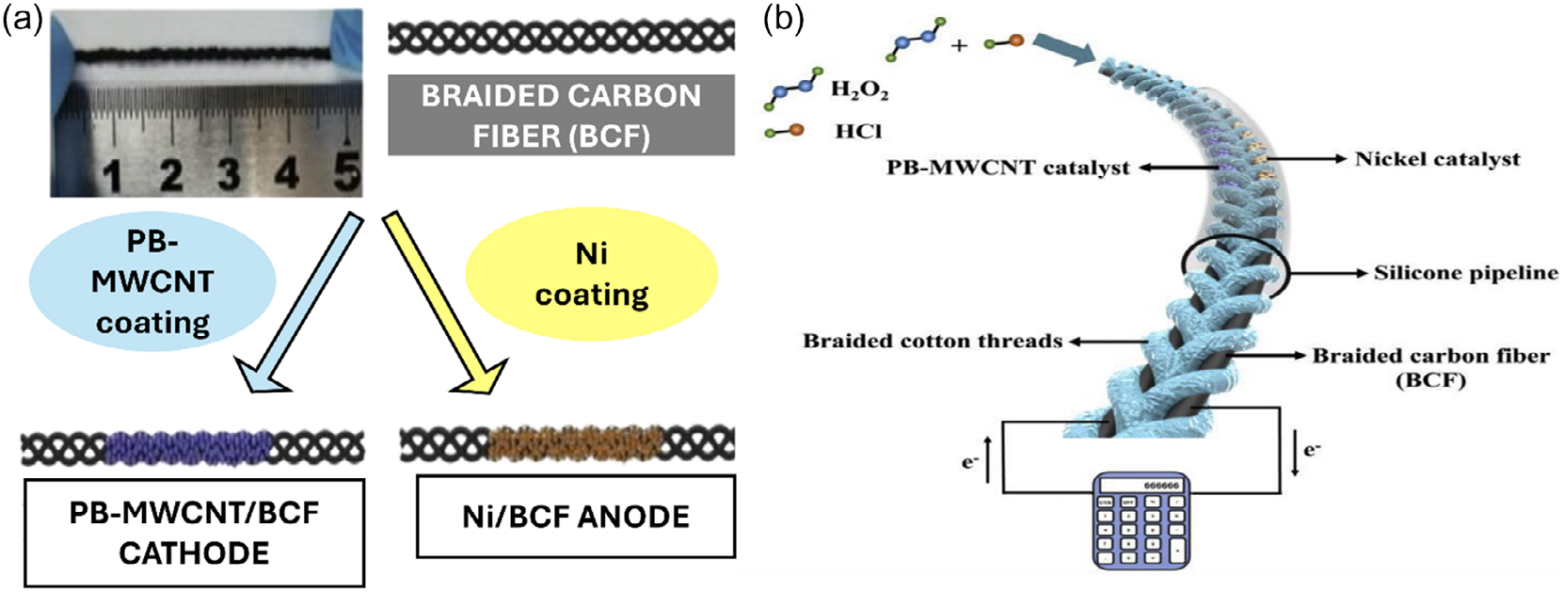
Schematics of a) electrode preparation and b) principle of the flexible on‐fiber H_2_O_2_ MFC. Reproduced with permission.^[^ ^26^ ^]^ Copyright 2022, Elsevier.

Transition metals such as nickel (Ni), iron (Fe), and cobalt (Co) have emerged as promising alternatives to noble metals in MLFCs due to their lower cost, natural abundance, and favorable electrochemical properties. Although their intrinsic catalytic activity is generally lower than that of platinum‐group metals, advances in material engineering—such as nanostructuring, doping, and hybridization—have significantly improved their performance, especially in systems designed for flexibility and portability. A notable contribution in this field is the work by Wang et al.,^[^ ^26^ ^]^ who developed a flexible on‐fiber MLFC using nickel‐coated braided carbon fibers as the anode and a Prussian Blue–multiwalled carbon nanotube (CNT) composite as the cathode. The FC uses hydrogen peroxide as both the fuel and the oxidant, simplifying the design by eliminating the need for separate chambers. The electrode architecture features a 3D flow‐through structure that increases surface area and improves access to electroactive sites, enhancing both HOR and ORR. The system also operates passively, with capillary forces and gravity driving the flow of reactants through the microchannels. This eliminates the need for external pumps and enables compact, energy‐efficient operation. The device was capable of powering a handheld calculator, demonstrating its potential for real‐world, low‐power applications. The electrode preparation and configuration of this system are shown in **Figure** **9**.

In addition to nickel‐based systems, transition metal chalcogenides and nitrogen‐doped carbon materials have gained attention for their high ORR selectivity and resistance to alcohol crossover. Catalysts such as RuSe and CoSe have shown the ability to suppress methanol oxidation while maintaining stable electrochemical performance.^[^ ^27^ ^]^ These features are particularly valuable in MLFCs using small organic fuels, where catalyst poisoning and instability remain major challenges. Their chemical resilience and selectivity contribute to improved efficiency and durability during prolonged operation.

Carbon‐based materials also play a central role in enhancing the performance of transition metal catalysts. Materials such as CNTs, mesoporous carbon (MC), and graphene are widely used as conductive supports or even as active electrocatalysts. For example, Fe_3_[Co(CN)_6_]_2_ supported on CNTs has been shown to improve hydrogen peroxide reduction and mechanical integrity.^[^ ^28^ ^]^ In a related study, wire‐shaped MLFCs were developed using biscrolled CNTs loaded with Fe_3_[Co(CN)_6_]_2_ at the cathode. The CNTs provided mechanical strength, flexibility, and high electrical conductivity, all of which are crucial for wearable applications. The biscrolling technique ensured uniform catalyst distribution while preserving the flexibility of the electrode. Together, these features enabled stable operation and effective integration into textile‐based systems.

Transition metal catalysts also offer important advantages in terms of mechanical adaptability, making them particularly suitable for flexible and lightweight power sources. Their compatibility with passive fluidic designs, resistance to mechanical fatigue, and tunable redox properties position them as strong candidates for next‐generation MLFCs. When combined with engineered carbon architectures, such as CNTs or MC, these systems can address persistent challenges like catalyst degradation and methanol crossover, which remain critical barriers to MLFC deployment.^[^ 29, 30 ^]^ More broadly, continued innovation in the design and integration of transition metal‐based catalysts—whether through structural modification, composite formation, or novel material pairing—represents a versatile and scalable approach for advancing the efficiency, durability, and real‐world viability of MLFCs.

#### 2.1.3 Hybrid Catalysts for MLFCs

Hybrid catalysts—combinations of noble metals, transition metals, and carbon‐based materials—have emerged as a highly effective strategy to enhance both the performance and durability of MLFCs. These materials are designed to harness the advantages of each component: the high catalytic activity of noble metals, the cost‐effectiveness and functional versatility of transition metals, and the conductivity and structural stability of carbon supports. Through careful integration, hybrid systems can address key limitations such as fuel crossover, catalyst poisoning, and low selectivity, while maintaining high reaction rates and long‐term stability under realistic operating conditions.

Among the most studied systems are multimetallic alloy catalysts, which combine noble and transition metals to enable multiple synergistic effects. A representative example is the Pt–Ru–Ni/C catalyst developed for methanol oxidation in acidic environments.^[^ ^31^ ^]^ In this system, nickel introduces redox‐active Ni(OH)_2_/NiOOH species that provide an additional catalytic pathway, operating in parallel with the classical bifunctional mechanism of Pt and Ru. This dual mechanism improves overall reaction kinetics while simultaneously enhancing CO tolerance, a key issue in alcohol‐fed systems. Additionally, the incorporation of Ni contributes to the structural robustness of the catalyst layer, reducing deactivation from adsorbed intermediates and extending operational life. These results highlight how ternary alloy systems can boost both activity and long‐term durability in compact MLFC platforms.

A similar approach has been applied to ethanol oxidation using a ternary Pt–Sn–Re alloy supported on MC.^[^ ^32^ ^]^ In this system, Sn facilitates water activation and intermediate oxidation, while Re contributes to the cleavage of C—C bonds, promoting more complete ethanol oxidation. The Pt–Sn–Re/MC (80:15:05) formulation demonstrated superior performance in both power density and current output compared to its monometallic and bimetallic counterparts. The MC support further contributed by providing high surface area, excellent conductivity, and uniform metal dispersion. The incorporation of Re was especially critical in reducing the accumulation of intermediates such as CO, leading to improved catalyst efficiency and resilience.

Building on these strategies, more advanced hybrid nanostructures have been developed to enhance performance under high fuel concentrations. For example, RuSex/C has demonstrated sustained ORR activity and excellent methanol tolerance, even in environments with significant fuel crossover.^[^ ^33^ ^]^ This material combines transition metal components with conductive carbon frameworks to improve both the kinetics of oxygen reduction and the stability of the catalyst layer. Their 3D architectures facilitate efficient mass transport and electron transfer, minimizing the impact of fuel crossover and contributing to higher overall efficiency under realistic operating conditions.

Taken together, these studies highlight the critical role of hybrid catalysts in addressing persistent limitations in MLFCs, such as intermediate poisoning, incomplete oxidation, and low fuel selectivity. By integrating the catalytic efficiency of noble metals with the structural and electronic advantages of transition metals and carbon‐based supports, these systems enable enhanced reaction kinetics, greater tolerance to alcohol fuels like methanol and ethanol, and stable operation within colaminar flow architectures. The integration of selective electrocatalysts is therefore essential to minimize fuel crossover effects and improve system efficiency.^[^ ^34^ ^]^ One of the primary challenges remains the competition between oxygen reduction and undesired alcohol oxidation at the cathode, which can significantly reduce overall performance.^[^ ^35^ ^]^ Future efforts should prioritize the refinement of catalytic formulations and electrode architectures to further enhance methanol tolerance and ensure stable operation under practical conditions.^[^ ^36^ ^]^ These developments will be central to advancing MLFCs toward practical implementation in portable, wearable, and decentralized energy systems.

## 3 Mixed‐Reactant MFCs

MRFCs operate by simultaneously introducing both the fuel and oxidant into a single reaction chamber, removing the need for a separator membrane. This streamlined design reduces system complexity and cost, making them attractive for compact and portable applications. However, unlike laminar systems where the separation of reactants is partially controlled by flow dynamics, MRFCs rely entirely on the intrinsic properties of the electrocatalysts to maintain spatial and chemical selectivity. As a result, electrocatalyst design plays a central role in MRFC performance.^[^ ^37^ ^]^ Catalysts must not only promote the desired oxidation and reduction reactions but also prevent cross reactions that would otherwise occur in the mixed environment. Selectivity is therefore critical to avoid mixed potentials, minimize energy losses, and ensure efficient fuel utilization.^[^ ^13^ ^]^ Recent works have demonstrated that improvements in catalyst composition and electrode configuration can significantly enhance performance, validating the potential of MRFCs for efficient and scalable energy generation.

### 3.1 Noble Metal‐Based Catalysts for MRFCs

Noble metals, particularly Pt and Pd, have long been central to FC technology due to their excellent catalytic activity and stability. In the context of MRFCs, where fuel and oxidant coexist in a single reaction zone, these materials continue to play a critical role. Their high electrochemical performance and resistance to degradation make them ideal candidates for selective electrocatalysis, which is essential in MRFCs where spatial or flow‐based separation is absent.

The concept of catalyst‐selective MRFCs was effectively demonstrated by Yu and Manthiram^[^ ^38^ ^]^ in a membraneless alkaline direct formate FC, where both fuel and oxidant were present in the same chamber. Using Pt/C and Pd/C electrodes, the system relied entirely on the intrinsic selectivity of the catalysts to enable distinct oxidation and reduction reactions without the need for a physical membrane. While their design was based on laminar flow, the underlying principle remains relevant to MRFCs: reaction specificity can be preserved through catalyst selectivity alone, offering a simpler and potentially scalable alternative to conventional FC configurations. This study demonstrated that noble metal catalysts could, under optimized conditions, effectively prevent crossover reactions and maintain stable operation, reinforcing the feasibility of MRFCs based solely on electrocatalytic control. However, despite their promise, this strategy has not yet reached wide‐scale adoption, likely due to lingering challenges in long‐term stability, integration, and upscaling.

Additionally, silver has been investigated as a strategy to enhance ORR selectivity in membrane‐free configurations. A clear example is the work by Abrego‐Martínez et al.^[^ ^39^ ^]^ By introducing a bilayer cathode architecture—consisting of a top Ag layer and a bottom Pt layer—the authors demonstrate that Ag acts as an effective ethanol barrier while allowing oxygen access to the underlying Pt sites. This configuration significantly suppresses ethanol oxidation at the cathode without compromising ORR activity, enabling stable operation even under high ethanol concentrations (3 M). The strategy exemplifies how catalyst‐layer engineering, rather than mere material substitution, can unlock new performance windows in MRFCs. The work further underscores the potential of rationally designed composite electrodes to decouple reactivity and selectivity challenges, particularly in mixed‐reactant systems. Importantly, this approach is compatible with planar microfluidic architectures and low‐cost fabrication methods, suggesting a scalable route for improving catalyst tolerance and fuel utilization.

Another study that leverages the favorable properties of silver—this time in combination with palladium—demonstrates how bimetallic catalysts can reduce the reliance on high‐load noble metals while maintaining strong electrocatalytic performance. In this work, Pd/Ag catalysts have shown excellent activity and durability in membrane‐free regenerative formate–oxygen FCs (MRFBFCs) operating with alkaline potassium formate and oxygen.^[^ ^40^ ^]^ The catalyst Pd/Ag was applied at both the anode and cathode, delivering efficient electrocatalysis while also improving resistance to catalyst degradation under harsh conditions. A major milestone of this work was the construction of a 19‐cell bipolar stack, which successfully powered an electric scooter—the first real‐world application of a MRFBFC system. The single cells achieved a peak power density of 4000 W m^−2^, demonstrating that efficient catalysis is possible even when fuel and oxidant are delivered in a single stream.

### 3.2 Transition Metal‐Based Catalysts for MRFCs

As MRFCs move toward more accessible and cost‐effective designs, transition metals have attracted growing interest as alternatives or complements to noble metal catalysts. Materials such as manganese, iron, cobalt, and nickel are abundant, relatively inexpensive, and offer tunable catalytic properties that make them promising for application in mixed‐reactant systems. However, because MRFCs lack a membrane or flow‐based separation mechanism, transition metal catalysts must provide not only high activity but also intrinsic selectivity to prevent undesired crossover reactions and preserve the spatial resolution of redox processes.

**Figure 10:**
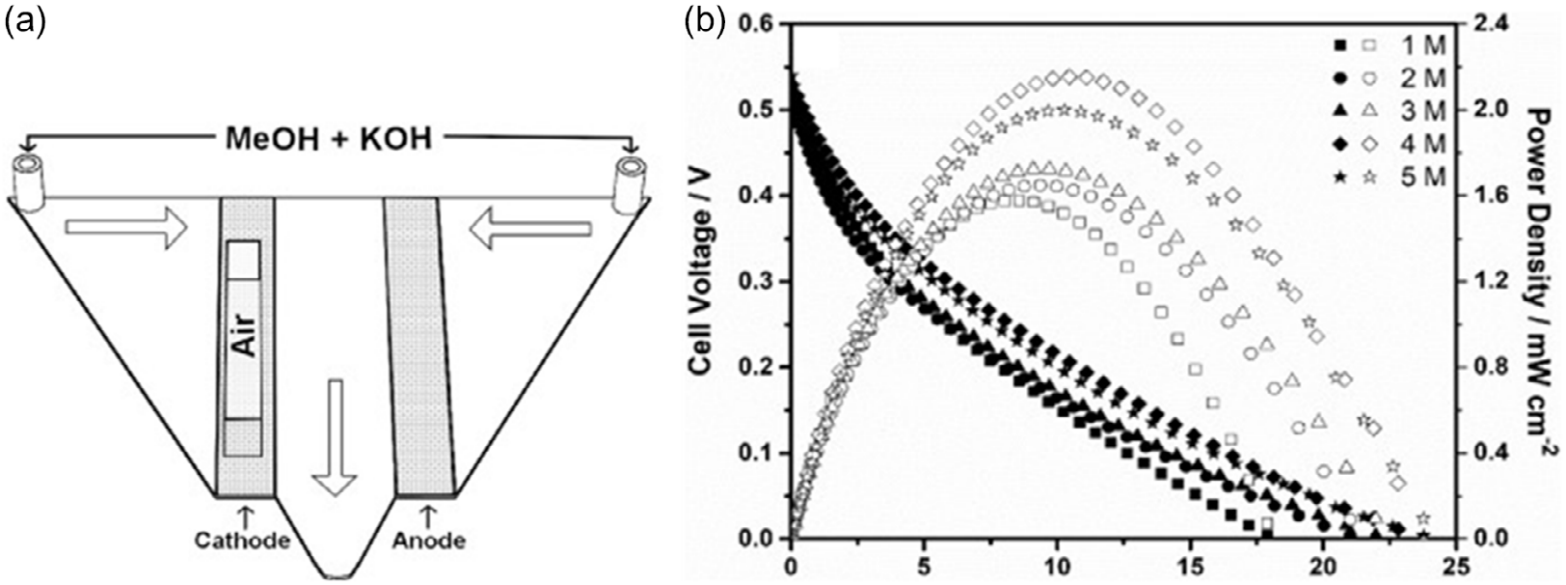
a) Schematic of the μ‐DMRFC testing system. b) Polarization curves (filled symbols) and power density curves (open symbols) recorded during passive operation of the μ‐DMRFC with different concentrations of MeOH in 0.5 M KOH under mixed‐reactant conditions. Reproduced with permission.^[^ ^23^ ^]^ Copyright 2019, Elsevier.

A notable example of this selective approach is the Mn_2_O_3_/Pt catalyst, which integrates manganese oxide with platinum to improve ORR activity while suppressing methanol crossover at the cathode.^[^ ^34^ ^]^ In this system, Mn_2_O_3_ acts as a selective barrier that permits oxygen transport to Pt catalytic sites while blocking larger methanol molecules, thereby enhancing cathodic efficiency in methanol‐rich environments. The catalyst was fabricated using pulsed laser deposition (PLD) to finely control surface morphology and layer structure, optimizing the balance between accessibility and selectivity. In the field of transition metal‐based catalysts, this Mn_2_O_3_/Pt catalyst is a notable advancement for MRFCs. Experimental evaluation was conducted using a microfluidic direct MRFC (μ‐DMRFC) fed with methanol concentrations ranging from 1 to 5 M in 0.5 M KOH. As shown in **Figure** **10**a, the μ‐DMRFC system features a compact architecture suited for passive operation. Figure 10b presents the resulting polarization and power density curves. Peak performance was achieved at a methanol concentration of 4 M, with a maximum power density of 2.16 mW cm^−2^, while slightly lower output (2.0 mW cm^−2^) was observed at 5 M, likely due to CO poisoning of the platinum surface during methanol oxidation. These results demonstrate the catalyst's ability to maintain efficient operation under harsh mixed‐reactant conditions, highlighting its potential for practical, low‐maintenance MRFC applications.

**Figure 11:**
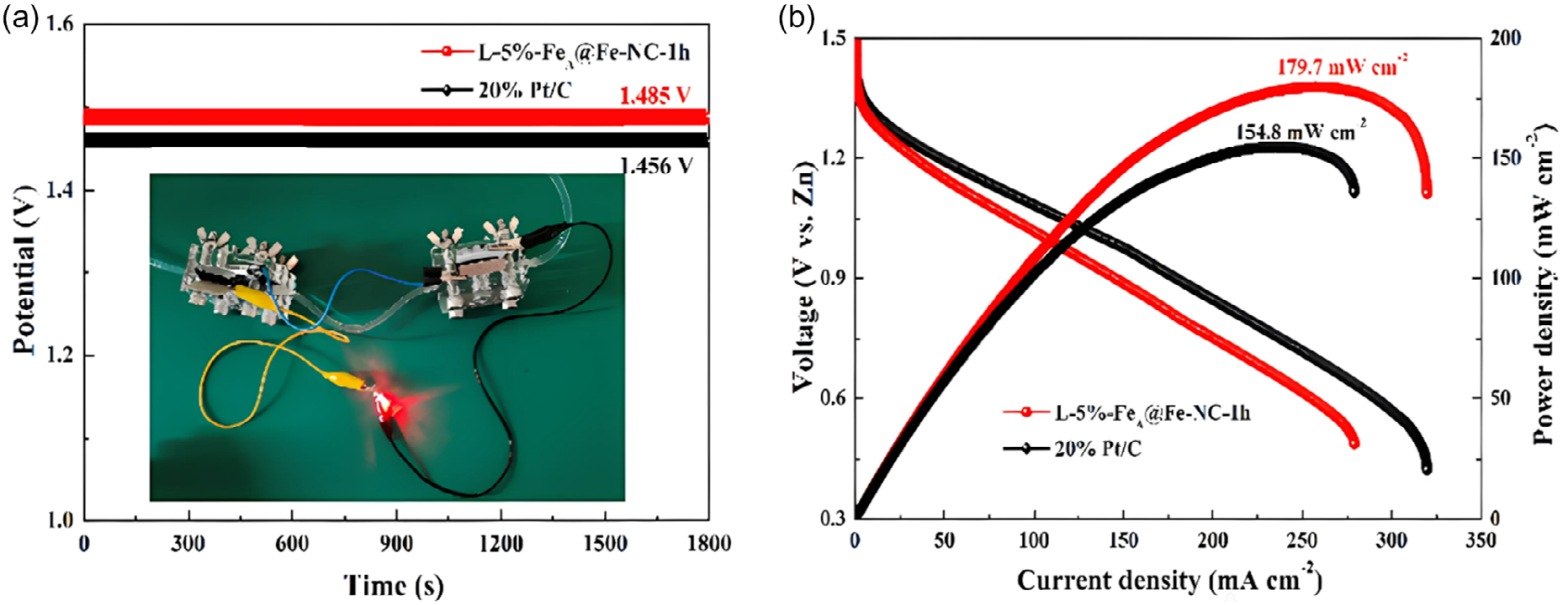
a) Open circuit potential (OCP) and b) discharge polarization curves of the L‐FeA@Fe‐NC and Pt/C catalyst. The FC based on L‐FeA@Fe‐NC shows a stable OCP and peak power density, demonstrating superior performance compared to the Pt/C‐based FC. Reproduced with permission.^[^ ^23^ ^]^ Copyright 2022, Elsevier.

A complementary strategy focuses on non‐noble hybrid catalysts that combine transition metals and engineered carbon materials. One of the most advanced examples is the L‐FeA@Fe‐NC‐1h system,^[^ ^41^ ^]^ composed of iron‐ and nitrogen‐doped carbon nanosheets synthesized via a self‐templating method. This material integrates Fe–N_*x*_ coordination sites—responsible for the ORR—with electrochemically inactive austenitic Fe nanoparticles that provide mechanical strength and corrosion resistance. The porous, 2D morphology facilitates efficient mass and charge transport, while interfacial boundaries improve local electron transfer and prevent Fe leaching. The catalyst shows strong ORR performance, excellent methanol tolerance, and long‐term stability, outperforming Pt/C in both open‐circuit potential and discharge polarization when tested in zinc–air batteries. As shown in **Figure** **11**a,b, the zinc‐air battery with the iron‐ and nitrogen‐doped carbon nanosheet‐based catalyst displays superior performance, with better stability and higher power density than the Pt/C‐based zinc‐air battery. These results underscore the value of rational architecture in designing nonprecious alternatives for MRFC cathodes.

In parallel, efforts to develop nonprecious metal catalysts have focused on enhancing the density and utilization of active sites in carbon‐supported structures. One such advancement involves a Fe–N‐doped MC catalyst synthesized via a solvothermal route.^[^ ^42^ ^]^ In this study, the interfacial energy between the FePc‐containing precursor solution and the porous carbon support was carefully tuned by selecting solvents with low surface tension, such as n‐hexane. This approach enabled deep infiltration of iron into the internal pore structure rather than surface deposition, resulting in a high density of uniformly distributed Fe–N_*x*_ and graphitic‐N active sites. The increased occupation of internal pores enhanced both the ORR activity and the resistance to crossover species such as methanol or formate, which are common challenges in mixed‐reactant environments. The resulting catalyst demonstrated excellent electrochemical stability and selectivity under fuel‐rich conditions, confirming the effectiveness of solvent–support interaction control as a strategy for catalyst design. These findings provide a scalable route for producing high‐performance, nonprecious metal catalysts specifically tailored for MRFC applications.

### 3.3 Hybrid Catalysts

Operating without membranes, MRFCs demand catalysts that can do more than just accelerate reactions—they must actively manage how and where those reactions occur. Since there is no membrane to separate half‐cell processes, hybrid catalysts must prevent crossover reactions and suppress undesired electrochemical pathways, all while maintaining efficient redox performance. To meet these demands, recent efforts have focused on multifunctional catalytic systems capable of providing spatial and chemical selectivity. In this section, the term hybrid catalyst refers to multicomponent systems that integrate different functional materials—such as noble metals, transition metal oxides, and engineered carbon supports—into a single catalytic structure. This design strategy seeks to exploit synergistic interactions among the components to overcome individual limitations such as poor conductivity, low selectivity, or chemical instability. As such, hybrid catalysts represent a promising approach to achieving the complex performance requirements of MRFCs through integrated material design.

A well‐established example of hybrid catalyst use in MRFCs is found in platinum‐based ternary systems for alcohol oxidation. For instance, Pt–Sn–Ru/C catalysts in acidic conditions for formaldehyde electrooxidation^[^ ^43^ ^]^ highlighted the potential role of Sn in enhancing surface oxophilicity, possibly aiding the desorption of reaction intermediates such as CO or formate, while Ru may promote the availability of OH groups at low potentials, supporting more efficient oxidation. Fine‐tuning the catalyst composition—particularly by minimizing the Sn content—was associated with improved catalytic performance. While the cell configuration remained conventional, the findings reinforced the importance of elemental synergy and fine control over atomic ratios in noble‐metal hybrid catalysts for MRFCs.

**Figure 12:**
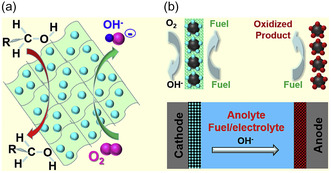
Schematic representation of the system proposed by Yu et al., showing a) a catalyst with selective activity and b) a membraneless alkaline DLFC operating under a catalyst‐selective principle. Reproduced with permission.^[^ ^44^ ^]^ Copyright 2016, Elsevier.

A more radical departure from conventional architectures came with the catalyst‐selective strategy proposed by Yu et al.,^[^ ^44^ ^]^ who introduced a catalyst‐selective strategy to eliminate the need for membranes in alkaline DLFCs (shown in **Figure** **12**). Two hybrid cathode catalysts were developed: MnNiCoO_4_ nanoparticles on nitrogen‐doped multiwalled CNTs (N‐MWCNTs) and NiCo_2_O_4_ on nitrogen‐doped graphene. These materials demonstrated high ORR activity under alkaline conditions, while remaining electrochemically inactive toward the oxidation of typical organic fuels such as methanol, ethanol, ethylene glycol, and glycerol. This intrinsic selectivity allowed the fuel and oxidant to coexist in the same reaction chamber without the need for laminar flow or a physical barrier. The system delivered high open‐circuit voltages and competitive current and power densities for all fuels tested, confirming that membrane‐free operation can be achieved through catalyst selectivity alone.

The concept was expanded in a follow‐up study by Yu and Manthiram,^[^ ^45^ ^]^ who demonstrated the versatility of this approach using potassium formate as fuel and Pd/C at the anode. Operating at temperatures up to 75 °C, the system showed consistent electrochemical performance across five different fuels. The study also addressed practical issues such as CO_2_ poisoning of the alkaline electrolyte, proposing strategies for long‐term operation and electrolyte maintenance. Together, these works transitioned the selective catalyst approach from proof‐of‐concept to a scalable, application‐oriented platform for MRFCs.

Further work with transition metal‐based hybrids includes the use of Prussian blue (PB) as a cathode catalyst in hydrogen peroxide MRFCs, where H_2_O_2_ acts simultaneously as fuel and oxidant in a fully symmetric configuration.^[^ ^46^ ^]^ This dual‐role design simplifies the system architecture by eliminating the need for separate reactant streams and complex flow control. PB, composed of iron‐centered cyanide‐bridged frameworks, demonstrates excellent electrocatalytic activity and chemical stability under acidic conditions. When supported on carbon, it achieves high durability and enhanced electron transfer, offering a low‐cost alternative to platinum for peroxide‐based systems. The hybrid design effectively combines the catalytic functionality of transition metals with the conductivity and surface area of carbon frameworks, enabling stable operation over extended periods. Importantly, the use of a single reactant for both electrodes not only reduces system complexity and cost but also provides a platform for compact and portable MFCs.

Finally, hybridization strategies have also enabled entirely new classes of MRFCs through the integration of photoactive components. One innovative example is a membrane‐free photocatalytic FC with dual photoelectrodes, designed to achieve simultaneous electricity generation and pollutant degradation.^[^ ^47^ ^]^ The system uses a vertically aligned nanorod array of WO_3_ coated with graphitic carbon nitride (g‐C_3_N_4_) at the photoanode, and Fe^3^^+^‐doped CuBi_2_O_4_ deposited on ITO at the photocathode. This configuration creates a Z‐scheme heterojunction, allowing for efficient light harvesting, directional charge separation, and improved redox performance under solar illumination. The photoanode drives the oxidation of organic contaminants, while the photocathode facilitates the ORR, ensuring balanced charge flow. The Fe doping at the cathode enhances band alignment, facilitating better photoelectron collection. Notably, the system operates without external bias or sacrificial agents, demonstrating a self‐sustaining photoelectrochemical platform. Both photoelectrodes contribute actively to the redox processes, driving the degradation of organic pollutants while generating usable electrical energy. This dual‐functionality system exemplifies how hybrid catalysts—engineered at both the chemical and electronic level—can expand the scope of MRFC applications beyond power generation, offering multifunctional devices for decentralized energy and environmental remediation (**Table** **1**).

**Table 1:** Recent performance parameters of membraneless laminar flow FCs (MLFCs) and mixed‐reactant MRFCs.

Fuel	Oxidant	Anode	Cathode	Current [mA cm^−2^]	Power [mW cm^−2^]	OCV [V]	References
MLFCs							
Aluminum	Air	Al	Pt/C	1053	690	2.2 (vs (Hg/HgO)	[20]
Methanol	O_2_	PtRu	Pt black unsupported	40	12	1.40 (vs Ag/AgCl)	[16]
H_2_O_2_	H_2_O_2_	Ni/BCF (nickel‐coated braided carbon fibers)	PB‐MWCNT/BCF (Prussian Blue multiwalled CNTs)	101.41	14.41	0.65 (vs Ag/AgCl)	[26]
H_2_O_2_	H_2_O_2_	CNT@Ni	CNT@Fe_3_[Co(CN)_6_]_2_	–	6.28	0.88 (vs Ag/AgCl)	[28]
H_2_O_2_	H_2_O_2_	Al	PB (lead(II) ferrocyanide)	6.16	1.43	0.98 (vs Ag/AgCl)	[23]
Ethanol	O_2_	Pt–Sn–Re/MC (80:15:05)	Pt/MC (100)	196.45	31.5	0.66 (vs Ag/AgCl)	[32]
H_2_O_2_	H_2_O_2_	Au@CF (electroplated)	Ni‐foam	0.011	0.8	≈0.55 (vs Ag/AgCl)	[25]
Glycerol	Air	Pd–Pt/C	Commercial Pt/C	≈7	1.6	0.88 (vs RHE)	[24]
Methanol	Perborate	Pt_50_Ru_40_Ni_10_/C	Commercial Pt/C	199.1	38.2	0.81 (vs Ag/AgCl)	[31]
Ethanol	O_2_	PtRu/C (50–50 at%)	Ag/Pt	54	10	0.75 V (vs RHE)	[39]
MRFCs							
MeOH	O_2_	Pt/CNTs	Mn_2_O_3_/Pt/CNT	24.2	2.00	0.54 (vs Ag/AgCl)	[34]
KHCO_2_	O_2_	Pd/Ag	Pd/Ag	1000	400	0.9	[40]
MeOH	O_2_	PtRu/C (50:50)	Mn_2_O_3_/Pt	150	28	0.8	[13]
HCOOK	O_2_	Pd/C	Pt/C	100	75	1.1 (vs SHE)	[38]
Ethanol	O_2_	PtRu/C	MnNiCoO_4_/N‐MWCNT	≈150	≈105	≈0.95 (vs RHE)	[44]
Ethylene glycol	O_2_	PtRu/C	NiCo_2_O_4_/N‐graphene	≈100	≈95	≈0.93 (vs RHE)	
Formate	Air	Pd on Ti mesh	Fe–N–C (Fe‐CBT/C6H14)	101.7	16.1	1.0 (vs RHE)	[42]
Oxytetracycline	Oxygen	WO_3_ nanorods on W, coated with g‐C_3_N_4_	ITO/CBFeO	0.62	0.11	0.90 (vs RHE)	[47]
Formaldehyde	Air	Pt–Sn–Ru/C (70:10:20)	Commercial Pt/C	230.2	36	0.63 (vs Ag/AgCl)	[43]
Methanol	Oxygen	Pt/C	FeA@Fe‐N‐C on porous carbon nanosheets	≈91	44.5	0.91 (vs RHE)	[41]
H_2_O_2_	H_2_O_2_	Ag foil or Ni mesh	Prussian Blue on carbon paper	≈1.22 (Ag), ≈1.55 (Ni)	≈0.8 (Ag), ≈1.55 (Ni)	≈0.53–0.6 (vs Ag/AgCl)	[46]

## 4 Current Approaches and Future Perspectives

The development of MFCs—whether laminar or mixed‐reactant—has evolved through a dynamic interplay between architectural simplification and catalyst innovation. Without a membrane to guide ion transport and isolate reactants, both FC configurations rely heavily on the precise selection and engineering of electrocatalysts to define reaction zones, suppress undesired processes, and maintain high performance. At the same time, advances in flow design and system integration have become essential to harness the full potential of these simplified architectures.

In MLFCs, the physical separation between fuel and oxidant is achieved through controlled laminar flow, forming a virtual membrane within a microchannel. In these systems, catalyst design must go hand in hand with fluid management. Materials such as Ni, Co, and Fe‐based oxides, as well as nitrogen‐doped carbon structures, have gained traction as efficient alternatives to noble metals. Supported on high‐surface‐area matrices like CNTs or MC, these catalysts offer enhanced kinetics, resistance to intermediate poisoning, and mechanical durability. Hybrid catalysts further expand this toolkit, integrating multiple functionalities to manage alcohol tolerance and improve mass transport.

Equally important is the fluidic architecture itself. Studies have demonstrated that channel configurations such as T‐shaped, F‐shaped, and bridge‐shaped geometries can significantly influence performance by optimizing reactant flow, minimizing ohmic losses, and improving the stability of the liquid–liquid interface. Dual‐electrolyte and double‐bridge designs have been shown to enhance mixing control and increase power output by as much as 171% compared to simpler layouts. Passive operation is often feasible in these systems, relying on capillary forces or gravity for fluid delivery—eliminating the need for external pumps and making MLFCs particularly attractive for portable and wearable applications.

MRFCs, by contrast, forgo any physical separation and instead rely entirely on catalyst selectivity to enable simultaneous oxidation and reduction in a shared environment. This places stringent demands on catalyst materials, which must be highly active for their target reactions while suppressing crossover effects and competing pathways. Hybrid materials have played a central role here as well. Mn_2_O_3_/Pt catalysts, for example, demonstrate methanol tolerance at the cathode, while Fe–N–C nanosheets localize ORR activity away from interfering fuel molecules. Prussian Blue‐based cathodes in peroxide‐fed MRFCs further showcase how non‐noble materials can stabilize redox reactions under challenging mixed‐reactant conditions. Looking forward, the next generation of MLFCs and MRFCs will depend on multifunctional catalysts that combine high activity, selectivity, and durability across diverse operating environments. In MLFCs, key priorities include reducing noble metal dependence, enhancing stability in flexible formats, and enabling miniaturized systems for use in wearables or diagnostic devices. In MRFCs, future strategies should focus on refining catalyst compositions to manage reactive fuel mixtures and suppress parasitic reactions without compromising performance. Promising approaches include redox‐active scaffolds, light‐assisted electrodes, and chemically selective interfaces capable of spatially guiding reactions.

Ultimately, progress in MFCs will come from integrating advanced electrocatalysts with efficient, pump‐free designs that are simple, scalable, and cost‐effective. Innovations like peroxide‐fed MRFCs with symmetric electrodes, MLFCs using flow‐through porous electrodes, and compact modular stacks are already demonstrating real potential for practical use. These devices point toward impactful applications ranging from environmental monitoring to portable electronics and emergency energy supply. As research in materials science, system design, and electrochemical engineering progresses, MFCs are expected to become simpler, more reliable, and easier to scale—combining efficient catalysts with thoughtful engineering to meet the energy needs of future technologies.

## 5 Conclusion

MFCs remain an emerging alternative to conventional systems, offering simplified architectures by eliminating the polymer membrane. This review has explored the latest advances driving their progress, particularly in the design of selective catalysts and the development of efficient system configurations. In laminar systems, functional separation is achieved through careful flow management, allowing fuel and oxidant to coexist without physical barriers. In mixed‐reactant systems, the solution relies on catalyst selectivity to promote desired reactions while suppressing undesired pathways. Recent developments include hybrid catalyst architectures combining noble and non‐noble elements, carbon‐based supports for durability, and fluidic designs that enhance fuel utilization and transport. Several prototypes have demonstrated promising power output and operational simplicity in small‐scale applications, confirming that these advances are more than theoretical. Moving forward, the field must shift from proof‐of‐concept to practical deployment by addressing long‐term material stability, reducing costs, and ensuring reliable integration. If these challenges are successfully addressed, these devices could offer a practical and efficient alternative for powering portable electronics, remote installations, and decentralized energy systems—particularly in contexts where traditional technologies are limited by complexity, maintenance needs, or cost.

## Conflict of Interest

The authors declare no conflict of interest.

## Author Contributions

**Ilaria Gamba** and **Gonzalo García:** conceptualization. **Meilyn Sanabria León** and **Maximina Luis‐Sunga:** methodology. **Maximina Luis‐Sunga**: formal analysis. **Meilyn Sanabria León** and **Nicolás Alejandro Sacco**: investigation. **Meilyn Sanabria León**: writing—original draft. **Maximina Luis‐Sunga** and **Nicolás Alejandro Sacco**: writing—review and editing. **Ilaria Gamba** and **Gonzalo García**: supervision. All authors have read and agreed to the published version of the manuscript.

## References

[1] M. A. Abdelkareem , K. Elsaid , T. Wilberforce , M. Kamil , E. T. Sayed , A. Olabi , Sci. Total Environ. 2021, 752, 141803. 32889267 10.1016/j.scitotenv.2020.141803

[2] N. P. Brandon , M. A. Parkes , Encyclopedia of Materials: Metals and Alloys, Elsevier, Oxford 2016, p. 377.

[3] A. G. Olabi , M. A. Abdelkareem , T. Wilberforce , E. T. Sayed , Renewable Sustainable Energy Rev. 2021, 135, 110026.

[4] M. A. Abdelkareem , E. T. Sayed , H. O. Mohamed , M. Obaid , H. Rezk , K.‐J. Chae , Prog. Energy Combust. Sci. 2020, 77, 100805.

[5] C. Xiao , T. He , L. Zhang , Flow Cells for Electrochemical Energy Systems: Fundamentals and Applications, Springer, Cham 2023, p. 21.

[6] W. Zhao , G. Xu , W. Dong , Y. Zhang , Z. Zhao , L. Qiu , J. Dong , Adv. Sci. 2023, 10, 2300550. 10.1002/advs.202300550 PMC10265069 37097627

[7] V. R. Kavya , K. Aparna , J. Electron. Comput. Netw. Appl. Math. 2023, 3, 29.

[8] L. Fan , Z. Tu , S. H. Chan , Energy Rep. 2021, 7, 8421.

[9] G. Xu , L. Yang , J. Li , C. Liu , W. Xing , J. Zhu , Adv. Sens. Energy Mater. 2023, 2, 100058.

[10] Q. Meyer , C. Yang , Y. Cheng , C. Zhao , Electrochem. Energy Rev. 2023, 6, 16.

[11] A. K. Shukla , R. K. Raman , K. Scott , Fuel Cells 2005, 5, 436.

[12] M. Tanveer , K.‐Y. Kim , Int. J. Energy Res. 2021, 45, 8536.

[13] A. M. Zuria , J. C. Abrego‐Martinez , S. Sun , M. Mohamedi , Renewable Sustainable Energy Rev. 2020, 134, 110045.

[14] B. Zhu , G. Meng , B.‐E. Mellander , J. Power Sources 1999, 79, 30.

[15] R. Ferrigno , A. D. Stroock , T. D. Clark , M. Mayer , G. M. Whitesides , J. Am. Chem. Soc. 2002, 124, 12930. 12405803 10.1021/ja020812q

[16] E. R. Choban , J. S. Spendelow , L. Gancs , A. Wieckowski , P. J. A. Kenis , Electrochim. Acta 2005, 50, 5390.

[17] E. Ramos , AIP Conf. Proc. 2005, 757, 207.

[18] I. H. Hanapi , S. K. Kamarudin , A. M. Zainoodin , U. A. Hasran , Int. J. Energy Res. 2019, 43, 8956.

[19] J. H. Oh , M. Tanveer , K. Y. Kim , Energies 2021, 14, 6973.

[20] L. Wang , R. Cheng , W. Wang , G. Yang , M. K. H. Leung , F. Liu , S. P. Feng , Electrochim. Acta 2021, 388, 138584.

[21] I. H. Hanapi , S. K. Kamarudin , A. M. Zainoodin , U. A. Hasran , Z. Zakaria , Micromachines 2023, 14, 1247. 37374832 10.3390/mi14061247 PMC10301901

[22] J. C. Shyu , S. H. Hung , Processes 2021, 9, 746.

[23] N. H. Park , J. Kim , Y. Ahn , Electrochim. Acta 2023, 446, 142106.

[24] D. Panjiara , H. Pramanik , Ionics 2020, 26, 2435.

[25] F. Zhu , G. Chen , A. Kuzin , D. A. Gorin , B. Mohan , G. Huang , Y. Mei , A. A. Solovev , J. Vis. Exp. 2023, 200, 65920. 10.3791/65920 37929967

[26] S. Wang , D. Ye , Z. Liu , X. Zhu , R. Chen , Q. Liao , Y. Yang , H. Liu , Int. J. Hydrogen Energy 2022, 47, 4793.

[27] J. H. Choi , C. M. Johnston , D. Cao , P. K. Babu , P. Zelenay , J. Electroanal. Chem. 2011, 662, 267.

[28] X. Zhou , X. Zheng , J. Xu , S. Dai , X. Wang , X. Hu , Y. Qiu , N. Yuan , J. Ding , Energy Technol. 2019, 7, 1900122.

[29] P. Nekooi , M. Akbari , M. K. Amini , Renewable Energy 2010, 35, 6392.

[30] D. C. Papageorgopoulos , F. Liu , O. Conrad , Electrochim. Acta 2007, 52, 4982.

[31] A. Arun , M. Gowdhamamoorthi , K. Ponmani , S. Kiruthika , B. Muthukumaran , RSC Adv. 2015, 5, 49643.

[32] M. Priya , B. Muthukumaran , RSC Adv. 2024, 14, 9646. 38525066 10.1039/d3ra06599e PMC10958457

[33] H. Cheng , W. Yuan , K. Scott , J. Power Sources 2008, 183, 678.

[34] J. C. Abrego‐Martínez , Y. Wang , A. Moreno‐Zuria , Q. Wei , F. M. Cuevas‐Muñiz , L. G. Arriaga , S. Sun , M. Mohamedi , Electrochim. Acta 2019, 297, 230.

[35] K. Scott , A. K. Shukla , C. L. Jackson , W. R. A. Meuleman , J. Power Sources 2004, 126, 67.

[36] D. Sebastián , A. Serov , K. Artyushkova , J. Gordon , P. Atanassov , A. S. Aricò , V. Baglio , ChemSusChem 2016, 9, 1986. 27376964 10.1002/cssc.201600583

[37] I. I. Riess , Funct. Mater. Lett. 2008, 1, 105.

[38] X. Yu , A. Manthiram , Appl. Catal., B 2015, 165, 63.

[39] M. J. Estrada‐Solís , J. C. Abrego‐Martínez , A. Moreno‐Zuria , L. G. Arriaga , S. Sun , F. M. Cuevas‐Muñiz , M. Mohamedi , Int. J. Hydrogen Energy 2019, 44, 18372.

[40] P. Forysinski , C. Oloman , S. Kazemi , T. Nickchi , A. Usgaocar , J. Power Sources 2019, 414, 366.

[41] X. Cao , J. Li , X. Dong , R. Song , X. Zhou , X. Wang , N. Yuan , J. Ding , J. Alloys Compd. 2022, 928, 166932.

[42] L. Lan , J. Li , Y. Yang , L. Zhang , L. Zhang , Q. Fu , X. Zhu , Q. Liao , Carbon 2022, 189, 240.

[43] K. Ponmani , S. M. Nayeemunisa , S. Kiruthika , B. Muthukumaran , Ionics 2016, 22, 377.

[44] X. Yu , E. J. Pascual , J. C. Wauson , A. Manthiram , J. Power Sources 2016, 331, 340.

[45] X. Yu , A. Manthiram , Energy Environ. Mater. 2018, 1, 13.

[46] S. A. Mousavi Shaegh , N. T. Nguyen , S. M. Mousavi Ehteshami , S. H. Chan , Energy Environ. Sci. 2012, 5, 8225.

[47] M. Huang , C. Zhou , R. Wen , J. Tian , W. Huang , H. Wei , J. Lu , J. Electrochem. Soc. 2022, 169, 026502.

